# Evaluation of the Long-Term Effect of Air Filtration on the Occurrence of New PRRSV Infections in Large Breeding Herds in Swine-Dense Regions

**DOI:** 10.3390/v4050654

**Published:** 2012-04-26

**Authors:** Scott Dee, Jean Paul Cano, Gordon Spronk, Darwin Reicks, Paul Ruen, Andrea Pitkin, Dale Polson

**Affiliations:** 1 Pipestone Veterinary Clinic, Pipestone, MN 56164, USA; Email: gdspronk@pipevet.com; 2 Boehringer-Ingelheim, St Joseph, MO 64501, USA; Email: jeanpaul.cano@boehringer-ingelheim.com (J.P.C); dale.polson@boehringer-ingelheim.com (D.P); 3 Swine Vet Center, St. Peter, MN 56082, USA; Email: dreicks@swinevetcenter.com; 4 Fairmont Veterinary Clinic, Fairmont, MN 56031, USA; Email: pruen@fmtvets.com (P.R); apitkin@fmtvets.com (A.P.); 5 University of Minnesota College of Veterinary Medicine, St. Paul, MN 55108

**Keywords:** porcine, reproductive, respiratory, syndrome, virus, air, filtration, breeding, herds

## Abstract

Airborne transmission of porcine reproductive and respiratory syndrome virus (PRRSV) is a risk factor for the infection of susceptible populations. Therefore, a long‑term sustainability study of air filtration as a means to reduce this risk was conducted. Participating herds (n = 38) were organized into 4 independent cohorts and the effect of air filtration on the occurrence of new PRRSV infections was analyzed at 3 different levels from September 2008 to January 2012 including the likelihood of infection in contemporary filtered and non-filtered herds, the likelihood of infection before and after implementation of filtration and the time to failure in filtered and non-filtered herds. Results indicated that new PRRSV infections in filtered breeding herds were significantly lower than in contemporary non-filtered control herds (*P* < 0.01), the odds for a new PRRSV infection in breeding herds before filtration was 7.97 times higher than the odds after filtration was initiated (*P* < 0.01) and the median time to new PRRSV infections in filtered breeding herds of 30 months was significantly longer than the 11 months observed in non-filtered herds (*P* < 0.01). In conclusion, across all 3 levels of analysis, the long-term effect of air filtration on reducing the occurrence of new PRRSV infections in the study population was demonstrated.

## 1. Introduction

Emerging and re-emerging diseases threaten the health and safety of animal populations around the world [1]. An example of an emerging disease of global significance is porcine reproductive and respiratory syndrome (PRRS) [2]. Recent pandemics of PRRS in China, also known as ‘‘blue ear disease” or ‘‘pig high fever disease” have resulted in losses of over 1 million pigs [3]. Since pork is the major meat product of China this shortage has more than doubled pork prices, contributed to the strongest inflation in a decade and precipitated intense social unrest [4,5]. In the United States, PRRS costs the swine industry over $500 million annually through elevated mortality, poor growth rates and additional costs of treatment and prevention [6]. The causative agent of PRRS, porcine reproductive and respiratory syndrome virus (PRRSV) is an RNA virus classified in the order Nidovirales, family *Arteriviridae*, and genus Arterivirus [7]. Following infection of naive swine, PRRSV undergoes rapid and constant change, challenging conventional methods of disease control such as vaccination [8,9,10]. Unfortunately, while extensive efforts have been made to eliminate PRRSV from infected populations, re-infection as a consequence of airborne spread of the virus is a frequent event [11]. Recently, evidence of long-distance airborne transport of PRRSV out to 4.7 km and 9.1 km has been published [12,13]; therefore, the filtering of incoming air to swine facilities has been proposed as a mean to reduce this risk [14]. This theory was recently tested under experimental conditions and over the course of a 4-year study period involving a model of a swine production region, airborne transmission of PRRSV to susceptible populations housed in filtered facilities was prevented 100% of the time [15]. 

As a result of these findings, air filtration has been rapidly applied to commercial swine production [16,17]. In order to measure its effect, pilot studies were conducted in large breeding herds in regions of southern Minnesota and northern Iowa, USA, with a high density of pigs. Although these studies generated promising results, they were limited by both sample size and duration of study period. Therefore, a long-term sustainability study was conducted to measure the effect of air filtration on reducing the risk of PRRSV infection in a large number of herds over an extended period of time. The study was based on the hypothesis that air filtration would significantly reduce the occurrence of new PRRSV infections when exposure of swine facilities to airborne PRRSV occurred. 

## 2. Results and Discussion

### 2.1. Experimental Design

The 38 participating herds selected for the study were organized in 4 independent cohorts according to time at risk in the control (no filtration) and treatment (filtration) groups. Characteristics of the 38 study herds are provided in Table 1 in accordance with their assigned cohort.

**Table 1:** Description of farm cohorts included in the study.

Cohort	Number of herds	Herd inventory/Mean number pig sites < 4.7 Km ^1^	Time at risk (months) ^2^	
			No filtration	Filtration
A	5	2746 sows/ 9 sites	0	40
B	5	3059 sows/ 6 sites	12	28
C	14	3557 sows/8 sites	24	16
D	14	3349 sows/7 sites	40	0

^1^ Number of inventoried sows in herd/number of pig sites located within 4.7 km of study herd; ^2^ Number of months that each cohort was evaluated pre- and post-filtration.

### 2.2. Summary of New PRRSV Infections

The study was conducted from September 1, 2008 to January 15, 2012. Throughout the study period, a total of 8 new PRRSV introductions occurred in the filtered herds while a total of 89 new introductions occurred in non-filtered herds. All infected herds exhibited clinical signs indicative of PRRS and PRRSV was present by polymerase chain reaction (PCR) in samples submitted to state diagnostic facilities. Nucleic acid sequencing indicated that in all cases, the open reading frame (ORF) 5 region of the new variants which entered the herds during the study period ranged in nucleotide heterology from 5 to 15% when compared with the sequences of historical PRRSV variants present in the herds when the study began.

#### 2.2.1. Likelihood of Infection in Contemporary Filtered and Non-Filtered Herds

New PRRSV infections in filtered breeding herds were significantly lower than in contemporary non-filtered control herds (*P* < 0.01). The odds for a new PRRSV infection in a non-filtered breeding herd was 8.03 (CI: 3.84–16.81) times higher than the odds in a filtered breeding herd. In filtered herds 0.01 new PRRSV infections were reported per herd month at risk or 0.17 infections per herd year at risk; whereas, in non-filtered herds 0.09 new PRRSV infections were reported per herd month at risk or 1.12 infections per herd year at risk (Table 2).

**Table 2:** Summary of new porcine reproductive and respiratory syndrome virus (PRRSV) infections in contemporary filtered and non-filtered breeding herds.

Group	Cohort	n	Enrolled	End of follow up	Time at risk/herd (months)	Total herd time at risk (months)	Number of cases
Filtered (treatment)	A	5	Sep 08	Jan 12	40	200	3
	B	5	Sep 09	Jan 12	28	140	1
	C	14	Sep 10	Jan 12	16	224	4
	Total (24)					564	8
Non filtered (control)	D	14	Sep 08	Jan 12	40	560	41
	B	5	Sep 08	Sep 09	12	60	9
	C	14	Sep 08	Sep 10	24	336	39
	Total (33)					956	89

#### 2.2.2. Likelihood of Infection in Equivalent Periods of Time before and after the Implementation of Air Filtration

**Figure 1:**
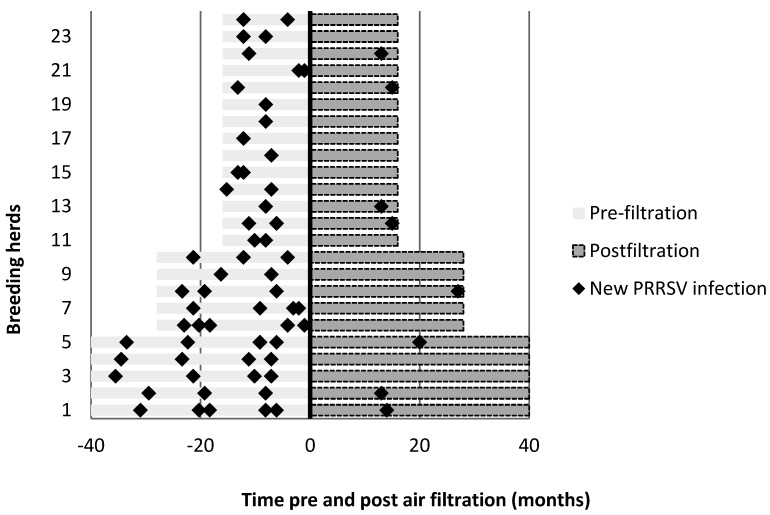
New porcine reproductive and respiratory syndrome virus (PRRSV) infections in breeding herds before and after the implementation of air filtration.

The distribution of new PRRSV infections in breeding herds in equivalent periods of time before and after the implementation of air filtration is summarized in Figure 1. Results are summarized in Table 3. The implementation of air filtration significantly reduced the occurrence of new PRRSV infections in breeding herds (*P* < 0.01). The odds for a new PRRSV infection in breeding herds before air filtration was 7.97 (CI: 3.77–16.85) times higher than the odds after air filtration was initiated. Before air filtration, 0.01 new PRRSV infections were reported per herd month at risk or 0.17 infections per herd year at risk; while 0.1 new PRRSV infections were reported per herd month at risk or 1.23 infections per herd year at risk. The mean time between new PRRSV infections by herd was 213 days prior to air filtration and 487 days after air filtration was implemented.

**Table 3:** Summary of new PRRSV infections before and after air filtration in breeding herds.

Group	Cohort	n	Enrolled	End of follow up	Time at risk/herd (months)	Total herd time at risk (months)	Number of cases
Pre-filtration (control)	A	5	May 05	Sep 08	40	200	20
	B	5	May 06	Sep 09	28	140	17
	C	14	May 07	Sep 10	16	224	21
	Total (24)					564	58
Post-filtration (treatment)	A	5	Sep 08	Jan 12	40	200	3
	B	5	Sep 09	Jan 12	28	140	1
	C	14	Sep 10	Jan 12	16	224	4
	Total (24)					564	8

#### 2.2.3. Time to Failure (New PRRSV Infection) in Filtered and Non-Filtered Herds

**Figure 2:**
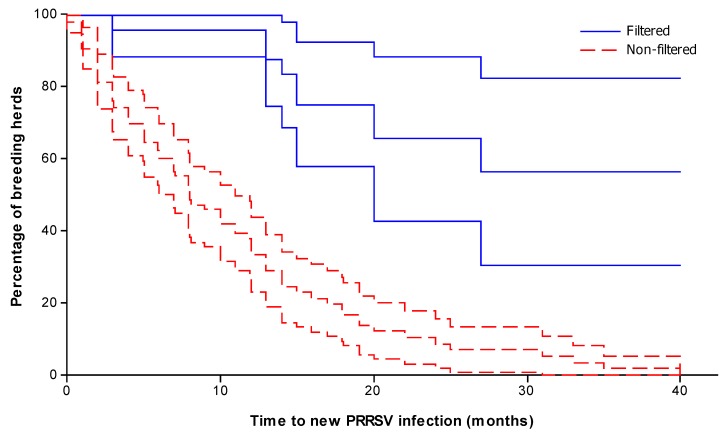
Kaplan-Meier curves for estimated survival functions for filtered and non‑filtered herds. Each estimate is accompanied by a point-wise 95% confidence interval.

The median time (months) to new PRRSV infections in filtered breeding herds of 30 months was significantly longer than the 11 months in non-filtered breeding herds during the study (*P* < 0.01). Results are summarized in Table 4 and Figure 2. Of note, the proportion of observations censored due to no new PRRSV introduction in the filtered farm data was 70.4% (19/27) whereas the proportion of censored observations in the non-filtered farm data was 19.4% (19/98). This suggests that, even though the period of evaluation was considerably longer for filtered farms (30 months) than non-filtered farms (11 months) a much higher proportion of filtered farms survived through the entire period of observation in contrast to the non-filtered farms, and a comparatively low number of observations contributed to the mean time to failure estimate for filtered farms. While for this portion of the analysis a question of possible temporal bias in the risk of site exposure could be raised (*i.e.*, the long-term ebb and flow of airborne PRRSV exposure of at-risk sites), the first analysis of contemporary cohorts may suggest that in these data a meaningful temporal bias was unlikely. 

**Table 4:** Summary of survival function parameters for filtered and non-filtered breeding herds.

Group	Observations		Mean time to failure (months)	Standard Error	95% CI	
	Censored	Uncensored			Lower	Upper
Filtered	19	8	29.99	3.06	24	35.98
Non-filtered	19	79	10.74	1.03	8.72	12.75

In today’s global swine industry, control, elimination and prevention strategies for PRRS are crippled by our inability to prevent the airborne spread of the virus among susceptible populations; therefore, it is critical to properly evaluate available strategies to mitigate said risk. Therefore, we designed a novel study which involved a large number of what were judged to be high-risk herds, an extended period of time and utilized multiple methods to critically analyze the data. The results obtained from the current analysis at three different levels are consistent and clearly indicate that under the conditions employed, air filtration significantly reduced the occurrence of new PRRSV infections in large breeding herds located in high pig density areas when compared to non-filtered cohorts. This multi-level approach allowed us to evaluate the study population from an integrated perspective that considered time at risk, year, season and time of intervention implementation. The authors are not aware of a previous report of PRRSV infections in breeding herds with this number of participant herds or follow up time. Event occurrence and time to event usually represent challenges for adequate analysis because the significant effect of time variables such as season and time at risk on the outcome. This is particularly true in databases that include partial year observations or artificially truncated follow up periods, where the probability of event occurrence might be greatly affected by the previously mentioned variables. However, the current database of participant herds allowed gathering information for an equivalent time before the follow-up period to perform a comparison before and after the implementation of the intervention in the herds. Also, the existence of contemporary control herds exposed to comparable conditions allowed evaluating the effect of filtration as the most important difference between those groups. 

## 3. Experimental Section

### 3.1. Breeding Herd Selection Criteria and Filter Installation

The study was conducted using protocols and procedures approved by the University of Minnesota Institutional Animal Care and Use Committee. Based on historical selection criteria [16,17], each candidate herd was required to have a breeding herd inventory of 2,400 sows or more. In addition, candidate herds needed to be surrounded by four or more growing pig sites within a radius of 4.7 km and candidate herds could not supply pigs to any of these surrounding sites. Finally, candidate herds had to have experienced a minimum of three external PRRSV infections over the past four years despite the use of industry standard biosecurity practices previously validated against known routes of direct and indirect spread of the virus [14]. As previously published, all the treatment herds used validated air filtration technologies (Camfill-Farr, Stockholm, Sweden or Clarcor, Jeffersonville, IN, USA) known to be compatible with negative-pressure ventilation systems [18]. Filters were installed in the attic of the breeding facility and/or as a filter bank placed externally to the facility’s evaporative cooling pad. Farms were allowed to only use previously validated filters at efficiencies of EU 9 (MERV 16) filters or EU 8 (MERV 14) filters which had been determined to be 95% and 75% efficient, respectively, at capturing particles 0.3 μm in diameter or larger [18]. 

### 3.2. Diagnostic Analysis of Study Herds

PRRSV status was monitored across all the herds on a monthly basis. During these visits, herds were assessed for clinical evidence of PRRS and production data were reviewed. In addition, blood samples were collected from 30 piglets from each herd at weaning and tested for the presence of PRRSV RNA using PCR [19] (Perkin-Elmer Applied Biosystems, Foster City, CA, USA). If positive, the open reading frame (ORF) 5 region of the virus from the sample in question was nucleic acid sequenced [20]. Nucleotide sequence phylogeny comparisons between the new sequence and historical sequences from the farm-specific database were then conducted. If clinical signs of PRRS were observed on the farm [10] and the percent nucleotide heterology between the sequence of the newly recovered PRRSV variant and farm-specific historical sequences exceeded published data regarding ORF 5 mutation rates following experimental passage through pigs (>0.5% per 367 days), it was concluded that a new PRRSV introduction had occurred [9].

### 3.3. Statistical Analysis

The effect of air filtration on the occurrence of new PRRSV infections in breeding herds was analyzed at three different levels in the study population, including the:

1. Likelihood of infection in contemporary filtered and non-filtered herds.
2. Likelihood of infection in equivalent periods of time before and after the implementation of air filtration.
3. Time to failure (new PRRSV infection) in filtered and non-filtered herds.

To compare the likelihood of infection with a new PRRSV isolate between filtered and non-filtered contemporary herds and between equivalent periods before and after air filtration was implemented in breeding herds, binary logistic regression was used to calculate the odds ratio and 95% confidence intervals (Minitab® 15, State College, PA, USA). The time that breeding herds were at risk was standardized by month. PRRSV infection (case) was defined as the detection of a new PRRSV isolate by PCR in the specific month. No more than one new PRRSV isolate was reported in a herd for each month, assuming a refractory period after an outbreak when no additional new cases could be detected. New PRRSV infection was included as the dependent variable and time at risk per herd (follow up period) as a random effect in the “contemporary” analysis but excluded from the “before and after” final model because the lack of significant contribution to the prediction of the outcome when tested in a multivariate logistic regression. Results were reported in odds ratios (OR) and 95% confidence intervals (CI). A value of *P* < 0.01 was considered statistically significant. To evaluate the time to new PRRSV infection in air filtered and non-filtered breeding herds, Kaplan-Meier (log rank) test was used to estimate the *P* value for the difference between groups (Minitab® 15, State College, PA, USA). Observed time to new PRRSV infection in months and 95% confidence intervals were represented in survival curves for both groups. A herd could be re-enrolled in the study after one month following the detection of a new PRRSV introduction. The observed time of the herds with no new PRRSV introduction during the follow up period was censored. A value of *P* < 0.01 was considered statistically significant.

## 4. Conclusions

This study provided new knowledge on the control of an economically significant disease of pigs. The execution of a strategy like air filtration carries an inherently high initial capital cost and substantial recurring operating cost, requiring justification for committing the capital required for implementation be based on an expectation of long-term airborne risk mitigation and consequence avoidance. While further evaluation of the long-term financial value characteristics of large-scale air filtration is needed, these findings may have immediate and far-reaching implications for animal well-being as it pertains to PRRSV and potentially other significant pathogens of livestock. For example, they may influence the future design of ventilation systems for agricultural buildings; thereby strengthening our ability to control and prevent other airborne diseases caused by RNA viruses such as H5N1 high pathogenic avian influenza and foot-and-mouth disease virus [21,22]. Finally, the ability of these systems to enhance the well-being of human populations by reducing the risk of spread of airborne agents, *i.e.*, SARS virus and influenza virus may expand their scope beyond livestock agriculture and significantly enhance their overall benefit.
